# The Role of Central Sensitization and Emotional Comorbidities in Temporomandibular Involvement Among Patients with Psoriatic Arthritis

**DOI:** 10.3390/life16040697

**Published:** 2026-04-21

**Authors:** José Antonio Blanco, Antonio Márquez, Esther Toledano, Rubén Queiro, Javier Martín-Vallejo, María José Fernández-Gómez, Carolina Chacón, Roberto Díaz-Peña, Daniel Martín, Cristina Hidalgo, María Dolores Sánchez, Moisés León González, Carlos Montilla

**Affiliations:** 1 Department of Surgery, Faculty of Medicine, University of Salamanca (USAL), 37007 Salamanca, Spain; jablancor@usal.es; 2 Oral and Maxillofacial Surgery Service, University Hospital of Salamanca (CAUSA), 37007 Salamanca, Spain; 3 Institute of Biomedical Research of Salamanca (IBSAL), 37007 Salamanca, Spain; amarvefisio@usal.es; 4 Department of Nursing and Physiotherapy, University of Salamanca (USAL), 37007 Salamanca, Spain; 5 Department of Rheumatology, San Carlos Clinical Hospital, 28040 Madrid, Spain; esthertoledano@hotmail.com; 6 Rheumatology Department, Hospital Universitario Central de Asturias—HUCA, 33011 Oviedo, Spain; rubenque7@yahoo.es; 7 Statistics Department, University of Salamanca (USAL), 37007 Salamanca, Spain; jmv@usal.es (J.M.-V.); mjfg@usal.es (M.J.F.-G.); 8 Department of Rheumatology, University Hospital of Salamanca (CAUSA), 37007 Salamanca, Spain; ccchacon@saludcastillayleon.es (C.C.); chidalgoc15@gmail.com (C.H.); 9 Immunogenetics Lab., Fundación Pública Galega de Medicina Xenómica, SERGAS, Grupo de Medicina Xenómica-USC, Instituto de Investigación Sanitaria de Santiago de Compostela (IDIS), 15700 Santiago de Compostela, Spain; roberto.diaz.pena@sergas.es; 10 Faculty of Health Sciences, Universidad Autónoma de Chile, Talca 3460000, Chile; 11 Primary Care Management, Valladolid West, 47012 Valladolid, Spain; dmartinh96@gmail.com; 12 Rheumatology Department, Hospital de Zamora, 49022 Zamora, Spain; mariol333@hotmail.com; 13 Department of Medicine, University of Salamanca (USAL), 37007 Salamanca, Spain; moileonglz@usal.es

**Keywords:** psoriatic arthritis, temporomandibular disorders, central sensitization, kinesiophobia, anxiety, pressure pain threshold, chronic pain, comorbidities

## Abstract

Background: Temporomandibular disorders (TMDs) are frequently underdiagnosed in patients with psoriatic arthritis (PsA), and the mechanisms underlying their development remain poorly understood. While inflammatory processes may contribute, central pain sensitization and psychological factors could play a significant role in TMD pathogenesis. Objective: The objectives of this study were to evaluate clinical characteristics, disease activity, psychiatric comorbidities, and pain processing mechanisms in PsA patients with and without TMD and to identify factors independently associated with temporomandibular involvement. Methods: This cross-sectional observational study included 190 consecutive PsA patients (CASPAR criteria) from a single tertiary center. Patients with fibromyalgia were excluded. TMD was assessed by maxillofacial specialists. Disease activity (cDAPSA), functional status (HAQ-DI), disease impact (PsAID-12), central sensitization (Central Sensitization Inventory, CSI), kinesiophobia (Tampa Scale for Kinesiophobia, TSK-11), pressure pain threshold (algometry), and emotional comorbidities (Hospital Anxiety and Depression Scale, HADS) were evaluated. An exploratory binary logistic regression identified a factor independently associated with TMD. Results: Twenty-five patients (13.1%) had confirmed TMD, with a significant female predominance (76% vs. 39%; *p* = 0.001). Only 24% of patients exhibited structural damage on orthopantomography. TMD patients showed higher CSI scores (52 vs. 32; *p* < 0.001), greater kinesiophobia (TSK-11: 30 vs. 23; *p* = 0.002), lower pressure pain thresholds (2.1 vs 2.7 kg/cm^2^; *p* = 0.03), and higher anxiety (HADS-A: 9 vs. 5; *p* = 0.001) and depression scores (HADS-D: 6.5 vs. 3; *p* = 0.001). TMD patients also exhibited worse functional status (HAQ-DI: 0.7 vs. 0.3; *p* = 0.001) and greater disease impact (PsAID-12: 4.8 vs. 2.9; *p* = 0.001). In multivariate analysis, central sensitization (OR: 1.1; 95%CI: 1.04–1.18; *p* = 0.001) and anxiety (OR: 1.2; 95%CI: 1.02–1.61; *p* = 0.02) were independently associated with TMD (Nagelkerke R^2^ = 0.48). Conclusion: TMD in PsA is associated with central sensitization and anxiety rather than mechanisms secondary to bone damage. These findings support a multidimensional approach incorporating screening for central sensitization and psychiatric comorbidities in PsA patients with temporomandibular symptoms.

## 1. Introduction

Psoriatic arthritis (PsA) is a chronic, immune-mediated inflammatory disease affecting approximately 17–30% of patients with psoriasis. It is characterized by a heterogeneous spectrum of clinical manifestations, including peripheral arthritis, enthesitis, dactylitis, axial involvement, and skin and nail disease [1,2,3]. Among the diverse articular manifestations of PsA, temporomandibular joint involvement has received comparatively little attention despite growing evidence of its clinical relevance. Despite the growing recognition of extra-axial and extra-appendicular joint involvement in PsA, the temporomandibular joint remains a notably understudied location, and its relationship with central pain processing mechanisms and emotional comorbidities has not been systematically investigated. Temporomandibular disorders (TMDs) encompass clinical conditions affecting the masticatory muscles and the temporomandibular joint (TMJ). Their classification comprises three main subcategories: condyle–disc complex derangements, structural incompatibility of the articular surfaces, and inflammatory disorders [4]. The etiology of TMD is multifactorial and includes accumulated microtrauma from parafunctional habits (bruxism and teeth clenching), sleep disturbances, and psychological factors [5]. The relationship between TMD and emotional factors is framed within a biopsychosocial model that recognizes the complex interaction between physical, psychological, and social aspects in the genesis and perpetuation of chronic pain [6,7]. Psychological stress plays a central role in this interrelationship, as symptoms such as skin pruritus, pain, and burning sensations in the oral mucosa, particularly when associated with stress states, can alter masticatory function through muscle hyperactivity. This phenomenon becomes pathological when associated with maladaptive processes that generate muscle or joint pain [7]. These findings underscore that pain in TMD frequently has a multifactorial origin, demanding a comprehensive therapeutic approach that addresses this complex interaction. This biopsychosocial framework becomes particularly relevant when TMD coexists with systemic inflammatory conditions, where peripheral inflammation and central pain mechanisms may converge to amplify temporomandibular symptoms. For this reason, chronic inflammatory joint diseases, such as PsA, should not be overlooked in comprehensive evaluations of TMD. For this reason, chronic inflammatory joint diseases, such as PsA, should not be overlooked in comprehensive evaluations of TMD. However, the TMJ represents a joint location that has been historically underdiagnosed and underestimated in the context of PsA, which has resulted in a notable scarcity of scientific evidence. The prevalence of TMD in patients with psoriasis and PsA is significantly higher than that observed in the general population [8]. An observational study by Crincoli et al. demonstrated that 68.8% of patients with psoriasis presented TMD symptoms, compared to only 24.1% of healthy controls, with this proportion reaching 80% in the subgroup with established PsA [9]. These findings were corroborated by Dervis et al., who identified a statistically significant increase in the clinical dysfunction index in patients with PsA compared to those with psoriasis without arthritis and healthy controls; muscle tenderness on palpation, joint sounds, and morning jaw stiffness or fatigue were the most frequent findings [10].

Nevertheless, few studies have specifically explored the association between TMD and PsA. Maranini et al. identified peripheral enthesitic involvement as a potential risk factor for the development of temporomandibular synovitis in patients with PsA [9]. Furthermore, Laškarin et al. documented an interrelationship between TMD and disease activity measured using the DAPSA index, observing an association with greater overall pain intensity [11]. Although these studies have established a clinical association between TMD and inflammatory disease burden in PsA, the underlying mechanisms driving temporomandibular pain in this population remain incompletely understood and likely extend beyond purely inflammatory pathways. The pain experience in PsA constitutes a complex and multifactorial phenomenon that transcends purely nociceptive mechanisms of inflammatory origin. The reduction in inflammatory markers through current therapies has not been consistently correlated with improvement in pain intensity in a substantial proportion of patients with PsA. This phenomenon has been linked to central sensitization (CS) and emotional comorbidities, particularly anxiety and depression [12,13]. Kinesiophobia, defined as excessive and irrational fear of movement derived from the catastrophic belief that it will cause pain or injury, constitutes another relevant psychological component in inflammatory rheumatic diseases. This phenomenon promotes avoidance behaviors that lead to muscle deconditioning, progressive disability, and intensification of pain perception, perpetuating a vicious cycle of pain and functional limitation [14,15].

Given this background, the primary objective of this study was to evaluate clinical characteristics, disease activity, functional capacity, psychiatric comorbidities, and pain processing mechanisms in patients with PsA, comparing those with and without mandibular involvement. As a secondary objective, we aimed to identify factors independently associated with the presence of temporomandibular involvement in this population.

## 2. Materials and Methods

Study Design and Participants

This cross-sectional observational study was conducted at a single tertiary center from 3 February 2025 to 15 September 2025. Consecutive patients fulfilling the following inclusion criteria were enrolled:

(a) Diagnosis of PsA according to the CASPAR classification criteria [16].
(b) Age ≥18 years.
(c) Provision of written informed consent.

The study protocol received approval from the Ethics Committee of the University Hospital of Salamanca (Code: CEIm: PI 2024 10 1749-TD; Approval date: 4 December 2024). The investigation adhered to the principles outlined in the Declaration of Helsinki.

Exclusion Criteria:

(a) Concomitant diagnosis of fibromyalgia [17].
(b) Assessed Variables

(a.1) TMD

Patients presenting with temporomandibular pain under follow-up by the Oral and Maxillofacial Surgery Department were included. All participants underwent a standardized clinical and radiological examination to assess temporomandibular involvement. TMD was diagnosed by the Oral and Maxillofacial Surgery Department following the Diagnostic Criteria for Temporomandibular Disorders (DC/TMD) Axis I protocol. This comprehensive clinical assessment included the evaluation of vertical and lateral mandibular ranges of motion, detection of joint sounds (clicking, crepitus, or crunching) via palpation and auscultation, and systematic palpation of the masticatory muscles and the TMJ capsule [18]. To complement the clinical examination and assess gross osseous structural integrity, a panoramic radiograph (orthopantomography) was obtained. This imaging modality allowed for the screening of condylar morphology and cortical erosions, as well as the exclusion of other dental or skeletal pathologies that could confound the diagnosis of PsA-related TMD. No specific soft tissue or inflammatory features were assessed with this technique, and ultrasound evaluation was not included in the study protocol. Joint pain upon provocation or palpation was recorded as a primary clinical marker of arthralgia or localized myalgia.

(a.2) Baseline Variables

The following demographic and clinical data were collected: age (years), sex, disease duration (years), and smoking habit (current, former, or never smoker). Current treatment was recorded, including conventional synthetic (csDMARDs), biologic (bDMARDs), and targeted synthetic (tsDMARDs) disease-modifying antirheumatic drugs. Disease phenotype was classified as peripheral (joint pain and swelling), axial (inflammatory back pain with radiographic sacroiliitis ≥ grade II or syndesmophytes), or mixed (fulfilling both definitions) [19,20]. Entheseal involvement was quantified through the modified Maastricht Ankylosing Spondylitis Enthesitis Score (mMASES) [21,22]. The presence of current or previous dactylitis was documented (yes/no). Skin involvement was graded with the Psoriasis Area and Severity Index (PASI), which scores erythema, induration, and desquamation across four body regions on a scale of 0–72, where higher values denote more extensive cutaneous disease [23].

(a.3) Disease Activity, Functional Status, and Impact

Disease activity in peripheral PsA was measured with the clinical Disease Activity index for Psoriatic Arthritis (cDAPSA), a purely clinical composite that sums tender (0–68) and swollen (0–66) joint counts, patient global disease activity VAS (0–10), and pain VAS (0–10), deliberately omitting CRP to distinguish it from the original DAPSA [24]. Disease activity was further categorized according to the cDAPSA cut-off values proposed by Schoels et al.: remission (≤4), low disease activity (>4 and ≤13), moderate disease activity (>13 and ≤27), and high disease activity (>27). Additionally, CRP levels (mg/dL) were recorded [24].

Physical function was captured with the Health Assessment Questionnaire–Disability Index (HAQ-DI), a 20-item tool covering eight functional domains scored from 0 to 3, with higher totals reflecting greater disability [25].

Overall disease burden was evaluated with the 12-item Psoriatic Arthritis Impact of Disease questionnaire (PsAID-12), a patient-reported measure that addresses daily activities, symptoms, and emotional well-being [26].

(a.4) Comorbidities

Psychological distress was screened with the Hospital Anxiety and Depression Scale (HADS), a 14-item questionnaire yielding two subscales (HADS-A, HADS-D; range 0–21 each) [27].

The fatigue subscale (FACIT-F), a 13-item measure validated in PsA, was scored from 0 to 52 on a 5-point Likert scale, with higher totals corresponding to lower fatigue burden [28].

Sleep quality was appraised with the Pittsburgh Sleep Quality Index (PSQI), which evaluates seven domains (subjective quality, latency, duration, efficiency, disturbances, medication use, and daytime dysfunction) over the preceding 30 days. Component scores (0–3 each) are summed into a global index (0–21), with values ≥6 denoting poor sleep [29].

Obesity: Adiposity was determined by calculating body mass index (BMI) [30].

(a.5) Central Sensitization

Central sensitization was assessed using the Central Sensitization Inventory (CSI), a 25-item self-administered questionnaire (Part A) with responses scored from 0 (never) to 4 (always), generating total scores between 0 and 100; elevated scores indicate a greater symptom burden associated with central sensitization [31].

(a.6) Kinesiophobia

Fear of movement was measured using the 11-item Tampa Scale for Kinesiophobia (TSK-11), a self-report instrument assessing fear of movement and (re)injury; each item is rated on a 4-point Likert scale (1 = strongly disagree to 4 = strongly agree), yielding a total score between 11 and 44, with higher values indicating greater kinesiophobia [32].

(a.7) Algometry

The pressure pain threshold (PPT) was determined using a Wagner FPX 25 digital algometer (Wagner Instruments, Greenwich, CT, USA) equipped with a 1 cm^2^ rubber tip. This device was previously calibrated by an external company (State of Connecticut Certification date: 3-15-19). Measurements were taken bilaterally at the trigger point (TP1) of the upper trapezius muscle, located midway between the acromion and the C7 spinous process. Pressure was progressively applied with the algometer display facing down (to avoid influencing the results) and perpendicular to the muscle surface until the patient indicated the first time the pressure sensation changed to pain or discomfort. Two consecutive measurements were taken with an interval of 30–60 s between them, and the arithmetic mean of both values was recorded as the final PPT, expressed in kg/cm^2^ or kPa (1 kg/cm^2^ ≈ 98 kPa) [33,34,35].

(b) Statistical Analysis

Quantitative variables are expressed as means and standard deviations (SDs) for normally distributed variables and as medians with interquartile ranges (IQRs) for non-normally distributed variables. Qualitative variables are expressed as numbers and percentages (n/%). Normal distribution was assessed using the Shapiro–Wilk test.

Comparisons between two groups were performed using Student’s *t*-test for normally distributed quantitative variables and the Mann–Whitney U test for ordinal or non-normally distributed quantitative variables. Comparisons among more than two groups were conducted using one-way ANOVA for normally distributed quantitative variables and the Kruskal–Wallis H test for ordinal or non-normally distributed quantitative variables.

An exploratory logistic model was performed with the presence/absence of TMD as the dependent variable, and variables reaching statistical significance in univariate analysis (*p* < 0.05) or previously described in the literature as associated with TMD were included as independent variables. Results are presented as odds ratios (ORs) with 95% confidence intervals (95% CIs). Model goodness of fit was evaluated using Nagelkerke’s R^2^ and the Hosmer–Lemeshow test. This multivariate model should be interpreted as exploratory given the modest sample size of TMD patients (n = 25); conclusions regarding independently associated variables are presented accordingly. To analyze the reliability and stability of the logistic regression results, the sample size was calculated based on the events per variable, the number of predictor variables, and the expected proportion [36]. Assuming 10 events per variable, 12 independent variables, and an expected proportion of 0.5, the sample size should be 240. The Variance Inflation Factor (VIF) was calculated to assess the presence of multicollinearity

Statistical analyses were performed using SPSS version 28 (IBM Corp, Armonk, NY, USA) for Windows.

## 3. Results

(a.1) Clinical Characterization of Temporomandibular Involvement.

Among the 190 PsA patients studied, 25 met the clinical and radiological criteria for confirmed TMD, representing a prevalence of 13.1%. The mean duration since TMD symptom onset was 3.4 years (SD: 5.0). Notably, 100% of the diagnosed patients reported localized joint pain during clinical maneuvers or palpation.

Regarding functional joint dysfunction, audible or palpable jaw sounds (including clicking and crepitus) were identified in seven patients (28.0%). Furthermore, an objective limitation of mandibular mobility (restricted mouth opening) was documented in six patients (24.0%). Seven patients exhibited condylar flattening (28.0%). Notably, none of the patients with PsA reported incident temporomandibular pain during the recruitment period.

(a.2) Demographic and Clinical Characteristics

Demographic characteristics and clinical parameters for the entire cohort and stratified by TMD status are shown in Table 1.

(a.3) Relationship Between TMD and Disease Activity, Function, and Impact

Patients with TMD exhibited higher disease activity, worse functional status, and greater disease impact. The remaining variables are displayed in Table 2.

When cDAPSA was categorized according to the cut-off values (remission ≤ 4, low >4–≤13, moderate >13–≤27, high >27), 14 patients with TMD (52%) exhibited moderate-to-high disease activity compared with 46 patients without TMD (28.9%; *p* = 0.007).

(a.4) Comorbidities

Patients with TMD demonstrated increased fatigue, elevated anxiety (HADS-A) and depressive symptom scores (HADS-D), poorer sleep quality, and higher body mass index. The results are presented in Table 3.

(a.5) Central Sensitization

The median CSI score for the entire cohort was 34 (22.2). Patients with TMD exhibited significantly higher CSI values: 52 (18) vs. 32 (21.5); *p* < 0.001.

(a.6) Kinesiophobia

The median TSK-11 score was 24 (13). Patients with TMD demonstrated higher TSK-11 values: 30 (13) vs. 23 (13); *p* = 0.002.

(a.7) Algometry

The PPT at the trapezius for the entire cohort was 2.6 (2.1) kg/cm^2^. Patients with TMD exhibited a lower pain threshold: 2.1 (1.8) vs. 2.7 (2.1); *p* = 0.03.

Multivariate Analysis

In binary logistic regression analysis with TMD (present/absent) as the dependent variable and sex (coded as 0 = female, 1 = male), enthesitis, Pain VAS, TJC, FACIT-F, HADS-A, HADS-D, PSQI, CSI, kinesiophobia, pain threshold and biological treatment (0 = No and 1 = Yes) as independent variables, the following results were obtained: CSI (*p* < 0.001, OR: 1.11; 95%CI: 1.05–1.18), HADS-A (*p* = 0.02, OR: 1.31; 95%CI: 1.02–1.61), and Nagelkerke R^2^: 0.50. The remaining variables were not statistically significant. Supplementary Table S1 presents odds ratio estimates and their corresponding confidence intervals to quantify the magnitude of the associations, addressing the potential impact of the small TMD sample size on statistical significance.

This multivariate model should be interpreted as exploratory due to the modest TMD + patient (n = 25) sample size; however, the consistent association of CSI and anxiety with TMD in both univariate and multivariate analyses strengthens the interpretation that these represent clinically meaningful markers. The model’s goodness of fit, as measured by Nagelkerke’s R, is 0.48 and the Hosmer–Lemeshow test was not statistically significant (Chi-square test = 4.19, df = 8, *p* = 0.839). No significant multicollinearity was observed among the independent variables, with all VIF values remaining below the threshold of 5 (see Table S2 in the Supplementary Materials).

## 4. Discussion

In our study, we found a relationship between TMD and central sensitization, hyperalgesia measured by pain threshold, and kinesiophobia; additionally, TMD was associated with emotional comorbidities.

Indeed, a decreased pain threshold in the trapezius was associated with the presence of temporomandibular pain. To the best of our knowledge, this association has not been previously published in patients with PsA. However, it has been described in patients without chronic inflammatory rheumatic disease. La Touche et al. demonstrated in their meta-analysis that the PPT was significantly lower in patients with TMD compared to controls [37]. From an indirect standpoint without the use of algometry, Fernández-de-las-Peñas et al. documented the existence of multiple active trigger points in the masticatory and cervico-scapular muscles (including the trapezius) in women with myofascial TMD [38]. Referred pain areas from neck trigger points were larger than those from masticatory muscles (*p* < 0.01), and the local and referred pain elicited from these trigger points reproduced the spontaneous pain pattern of TMD [38] to peripheral sensory and nociceptive stimuli, the hyperexcitability of dorsal horn spinal neurons, and the development of hyperalgesia and allodynia [11,39]. However, given the absence of advanced imaging or specific assessment of soft tissue and intra-articular inflammatory changes, these findings should be interpreted as clinical manifestations of pain processing rather than direct evidence of structural pathology. Nevertheless, this does not exclude a potential contribution of soft tissue alterations to the clinical picture. These observations reflect the complex interplay between peripheral nociceptive input, soft tissue alterations—such as capsular distension, synovial inflammation, or disc displacement—and central sensitization mechanisms, including hyperexcitability of dorsal horn spinal neurons and the development of hyperalgesia and allodynia [11,39]. This process may be intrinsic to PsA, as patients with concomitant fibromyalgia were excluded, and suggests that the symptomatic burden of TMD may arise from both peripheral inflammatory and soft tissue changes, in addition to central pain amplification, rather than being solely explained by bone damage.

The systematic use of panoramic radiography provided a reliable screening of the condylar osseous status across the entire cohort. While orthopantomography lacks the soft-tissue sensitivity of MRI for detecting early joint effusion or disc displacement, its integration with the DC/TMD clinical protocol allowed us to differentiate between structural destruction and functional pain. In line with this, Maranini et al. recently demonstrated in a large cohort of 272 patients with TMD symptoms that erosions were the only ultrasound finding significantly associated with TMJ symptoms (OR 2.26, 95% CI 1.06–4.84, *p* = 0.035), reinforcing the clinical relevance of structural bone damage—whether detected by ultrasonography or orthopantomography—as a key marker in the evaluation of temporomandibular involvement in rheumatic patients [40]. The finding of universal joint pain (100%) despite a low rate of radiographic mobility limitation (24%) suggests that TMD in psoriatic arthritis may be influenced by the interplay of inflammatory and neurobiological sensitization processes, although the cross-sectional nature of our study precludes establishing directionality. These observations are compatible with the hypothesis that the burden of TMD in PsA may be more closely aligned with central pain amplification (CSI) and psychological distress than with static radiographic damage alone.

However, kinesiophobia—a psychological component that perpetuates the cycle of chronic pain and disability through fear avoidance, causing patients to develop catastrophic beliefs about pain and engage in movement avoidance behaviors—was related to TMD. To the best of our knowledge, this relationship has not been found in patients with chronic inflammatory joint diseases. In patients with TMD, Marciniak et al. documented that functional limitations in TMD patients increase proportionally with the level of kinesiophobia (r = 0.621; *p* < 0.001) [41]. This process has also been correlated with signs of altered central processing, which is linked to impaired descending pain modulation and distal hyperalgesia [41]. The systematic review by Gui et al. on chronicity factors in TMD concluded that psychological distress (somatization, depression, fear of pain, fear of movement, and catastrophizing) and pain amplification characteristics (hyperalgesia and allodynia) contribute to the development of chronic TMD and that these factors interact with each other, complicating pain management [42]. In the same vein, Kim et al. concluded that fear of movement may play an important role in masticatory deterioration and that patient-reported outcomes (pain perception, catastrophizing, and kinesiophobia) appear to have greater influence on mandibular function than psychological distress (depression and anxiety) [43].

In contrast to those findings, in our study we found that anxiety was independently associated with TMD (HADS-A: *p* = 0.02, OR: 1.2; 95%CI: 1.02–1.61). These results are consistent with the longitudinal OPPERA project study, in which several psychological variables (including anxiety) predicted a higher risk of first-onset painful TMD, independent of somatic factors or baseline pain sensitivity. Similarly, a recent observational study classified TMD patients into two subgroups: those with central sensitization (TMD-CS) and those with predominantly nociceptive pain (TMD-nCS). In multiple regression analysis, anxiety was the only psychological factor moderately and significantly associated with greater pain intensity in both groups (TMD-CS: β = 0.468; *p* < 0.001 and TMD-nCS: β = 0.5087; *p* = 0.014) [44].

Although the cross-sectional design does not allow us to establish directionality, the association between temporomandibular involvement and greater psychiatric burden could hypothetically be explained by multiple bidirectional mechanisms: (1) chronic orofacial pain generates significant emotional distress; (2) anxiety promotes parafunctional behaviors such as bruxism, which in turn aggravates temporomandibular dysfunction; (3) both depression and anxiety amplify pain perception through modulation of descending inhibitory pathways; and (4) the systemic inflammation characteristic of PsA may promote neuroinflammation and alterations in monoaminergic neurotransmission associated with mood disorders [7,45].

In the same context of pain hypersensitivity and central sensitization, we found that patients with mandibular involvement had significantly higher disease activity, assessed by cDAPSA (15 vs. 10; *p* = 0.001), related to the presence of a greater number of tender joints (2 vs. 1; *p* = 0.01) and higher scores on visual analog scales for pain (6 vs. 3; *p* = 0.002) and patient global activity (6 vs. 4; *p* = 0.01). These findings are consistent with previous studies demonstrating a correlation between temporomandibular involvement and the extent/severity of peripheral joint involvement in PsA [4].

Furthermore, we found a significant association between peripheral enthesitis and mandibular involvement (median of 2 enthesitic points vs. 0; *p* = 0.009), which is particularly relevant from a pathophysiological perspective. These results are consistent with the findings in the study by Maranini et al., which demonstrated through multivariate analysis that peripheral enthesitic involvement represented a significant risk factor for the development of temporomandibular synovitis (*p* = 0.01), suggesting an enthesitic phenotype that predisposes to involvement of this joint [9].

Taken together, these findings highlight that TMD pain in PsA is not attributable to a single mechanism. Active peripheral inflammation—reflected by higher disease activity scores, tender joint counts, and enthesitis—and mechanical factors clearly contribute to temporomandibular symptoms. However, the independent association of central sensitization (CSI) and anxiety with TMD in the multivariate model, after adjusting for these inflammatory markers, suggests that central pain amplification represents an additional and distinct contributor rather than the sole driver of temporomandibular pain. This interpretation is further supported by the clinical–radiological paradox observed in our cohort: while 100% of TMD patients reported joint pain, only 24% exhibited structural radiographic changes, indicating that the symptomatic burden substantially exceeds what can be explained by mechanical damage alone. Therefore, we propose that TMD in PsA may result from the convergence of peripheral inflammatory nociception, enthesitis-driven mechanical stress, and superimposed central sensitization, with anxiety potentially acting as a modulatory factor that amplifies pain perception across all three pathways. However, it should be noted that, in the absence of advanced imaging techniques such as ultrasound or MRI capable of evaluating synovial and soft tissue involvement, these proposed mechanisms cannot be directly confirmed and should be interpreted as clinically inferred rather than structurally demonstrated. However, given the cross-sectional design, these associations should be interpreted with caution, and prospective longitudinal studies incorporating advanced imaging modalities are needed to elucidate the temporal and causal relationships between these variables.

The female predominance observed in the group with mandibular involvement (76% women versus 39% in the group without involvement; *p* = 0.001) coincides with findings in the literature on TMD of non-inflammatory origin, where women present higher prevalence and symptomatic severity. This sexual dimorphism could be explained by hormonal factors (estrogen receptors in the TMJ that modulate ligament laxity), differences in pain perception and communication, and a higher prevalence of psychiatric comorbidities in females [46,47].

Disease duration did not differ between groups (8 vs. 10 years; *p* = 0.5), suggesting that central sensitization in PsA may be more closely associated with current disease activity and psychological factors than with cumulative disease exposure.

The results of the present study have clinical implications: they underscore the need for systematic TMJ evaluation in patients with PsA, particularly in those with a female profile, higher disease activity, prominent enthesitic involvement, or emotional comorbidities.

Likewise, our findings support the incorporation of screening tools for CS and psychiatric comorbidities (HADS) in the routine evaluation of patients with PsA, especially in those with painful symptoms disproportionate to objective inflammatory signs or with suboptimal response to biological treatment. Furthermore, this suggests that, in addition to controlling systemic inflammatory activity, the management of temporomandibular involvement in PsA could benefit from incorporating interventions targeting central sensitization mechanisms and psychiatric comorbidities. Available evidence indicates that cognitive–behavioral therapies, graded exercise programs, pain neuroscience education, and, in selected cases, psychopharmacological treatment could complement the conventional therapeutic armamentarium [48,49].

The present study has some limitations that should be considered when interpreting the results. The cross-sectional design precludes establishing causal relationships between the analyzed variables. The relatively small size of the group with mandibular involvement (n = 25) limits the statistical power to detect weak associations. The evaluation of mandibular involvement was based on clinical criteria (DC/TMD Axis I) complemented by panoramic radiography, without confirmation by advanced imaging techniques (magnetic resonance imaging, ultrasonography), which could underestimate subclinical involvement, particularly early synovitis, disc displacement, or capsular widening. While MRI remains the gold standard for detecting early inflammatory changes such as joint effusion, synovitis, or internal disc derangement, and would have provided superior soft-tissue characterization, its systematic application in a cohort of 190 patients poses significant feasibility, cost, and accessibility limitations. We therefore prioritized panoramic radiography (orthopantomography) as a reliable, low-radiation screening tool to assess gross condylar morphology and exclude confounding dental or skeletal pathologies. Future studies should incorporate MRI or ultrasonography to provide a more comprehensive characterization of TMJ involvement in PsA, particularly for detecting soft tissue changes that may precede radiographic bone damage. The single-center nature of the study could limit the generalizability of results to other populations. Additionally, enthesitis was assessed using the mMASES, which, although validated and widely used in both axial and peripheral spondyloarthritis including PsA, primarily evaluates axial entheseal sites. The use of the Leeds Enthesitis Index (LEI), specifically developed for PsA and focused on peripheral sites, as a complementary tool could have provided additional characterization of entheseal involvement in this population [50]. This model should not be considered a predictive model due to the low number of TMD cases, which could lead to overfitting, but rather as an exploratory model aimed at determining the adjusted association between clinical variables and the presence or absence of TMD. Furthermore, the small cohort of TMD patients limits the study’s statistical power, potentially resulting in Type II errors where true associations remain undetected despite their clinical relevance.

As a strength, this is the first study to jointly analyze the triad of pain threshold–central sensitization–kinesiophobia and various comorbidities, as well as their relationship with TMD in patients with PsA.

## 5. Conclusions

Our findings reveal a complex, multidimensional landscape of temporomandibular involvement in psoriatic arthritis, characterized by a striking clinical–radiological paradox. The maxillofacial assessment showed that, while joint pain was a universal denominator among affected patients, only a minority exhibited structural mobility limitations. This suggests that TMD in the context of PsA represents a unique phenotype where early synovitis or functional myalgia precedes—and often overshadows—irreversible bone involvement. Ultimately, our study challenges the traditional reliance on static imaging techniques. While panoramic radiography remains an essential baseline screening tool, it frequently fails to capture the true symptomatic burden of the disease. We therefore advocate for a diagnostic shift toward international DC/TMD clinical standards and a therapeutic transition toward a multimodal approach. Effective management of TMD in PsA may benefit from integrating maxillofacial expertise with neuromodulatory and psychological interventions, addressing central sensitization mechanisms that may significantly contribute to the patient’s clinical reality. Nonetheless, these conclusions should be interpreted with caution given the exploratory nature of the multivariate model; the modest number of TMD cases (n = 25) may limit statistical power, increase susceptibility to overfitting, and affect the stability of the estimated effects.

## Figures and Tables

**Table 1:** Demographic and clinical characteristics stratified by TMD status.

Variable	All (n = 190)	TMD (n = 25)	No TMD (n = 165)	*p*
Age	55 (13)	52 (18)	56 (13)	0.1
Sex, N—male/female	107/83	6/19	101/64	0.001
Years since onset (years)	10 (9)	8 (10)	10 (9)	0.5
Smoking status				0.4
Smoker	46	4	42	0.4
Former smoker	83	9	74	0.5
Non-smoker	61	12	49	0.1
Conventional synthetic DMARDs N (yes/no)	132/58	15/10	117/48	0.1
Methotrexate	103	13	90	0.03
Sulfasalazine	21	2	19	0.03
Leflunomide	8	0	8	
tsDMARDs or bDMARDs, N (yes/no)	74/116	17/8	57/108	0.09
TNFa inhibitors	49	8	41	0.4
IL17 inhibitors	10	4	6	0.02
Ustekinumab	4	2	2	0.08
Guselkumab	5	1	4	0.5
iJAKs	2	0	2	
Apremilast	4	2	2	0.08
Clinical presentation, N				0.8
Peripheral	155	21	134	0.9
Mixed	29	3	26	0.7
Axial	6	1	5	0.5
mMASEs	0 (2)	2 (5)	0 (2)	0.009
Dactylitis (yes/no)	40/150	6/19	34/131	0.6
PASI	1 (2.4)	1 (2)	1 (2.4)	0.5

Abbreviations: DMARD: disease-modifying antirheumatic drug; tsDMARD: targeted synthetic disease-modifying antirheumatic drug; bDMARD: biologic disease-modifying antirheumatic drug; mMASEs: modified Maastricht Ankylosing Spondylitis Enthesitis Scoring; PASI: Psoriasis Area and Severity Index. Continuous variables are presented as mean ± SD or median (IQR) as appropriate; categorical variables are expressed as n (%).

**Table 2:** Disease activity, functional status, and disease impact.

Variable	All (n = 190)	TMD (n = 25)	No TMD (n = 165)	*p*
Pain VAS	4 (4)	6 (3)	3 (4)	0.001
Activity VAS	4 (5)	6 (4)	4 (4)	0.01
SJC	1 (1)	2 (2)	1 (1)	0.2
TJC	1 (2)	2 (5)	1 (2)	0.01
cDAPSA	11 (8.7)	13.2 (9.2)	10.6 (9.3)	0.09
CRP (mg/dL)	0.2 (0.4)	0.1 (0.2)	0.2 (0.4)	0.1
HAQ-DI	0.5 (0.9)	0.7 (0.6)	0.3 (0.8)	0.001
PsAID-12	3.4 (3.7)	4.8 (2.9)	2.9 (3.7)	0.001

Abbreviations: VAS: visual analogue scale; SJC: Swollen Joint Count; TJC: Tender Joint Count; cDAPSA: clinical Disease Activity Index for Psoriatic Arthritis; CRP: C-reactive protein; HAQ: Health Assessment Questionnaire; PsAID: Psoriatic Arthritis Impact of Disease. Continuous variables are presented as mean ± SD or median (IQR) as appropriate; categorical variables are expressed as n (%).

**Table 3:** Comorbidities and psychological symptoms stratified by TMD status.

Variable	All (n = 190)	TMD (n = 25)	No TMD (n = 165)	*p*
FACIT-F	40 (18.5)	30.5 (12.7)	41 (17)	0.001
HADS-A	5 (4)	9 (7.5)	5 (5)	0.001
HADS-D	3 (6)	6.5 (7.7)	3 (5)	0.001
PSQI	7 (8)	9 (7)	6 (7)	0.003
BMI (kg/m^2^)	26.7 ± 4.6	25.5 ± 5.4	27 ± 4.4	0.1

Abbreviations: FACIT-F: Functional Assessment of Chronic Illness Therapy—Fatigue; HADS-A: Hospital Anxiety and Depression Scale—Anxiety; HADS-D: Hospital Anxiety and Depression Scale—Depression; PSQI: Pittsburgh Sleep Quality Index; BMI: body mass index. Continuous variables are presented as mean ± SD or median (IQR) as appropriate; categorical variables are expressed as n (%).

## Data Availability

The data presented in this study are available upon request from the corresponding author.

## Supplementary Materials

The following supporting information can be downloaded at: https://www.mdpi.com/article/10.3390/life16040697/s1, Table S1: Coefficients of logistic Model. Table S2: Diagnosis of multicollinearity using the Variance Inflation Factor (VIF) for the model variables.

## Abbreviations

The following abbreviations are used in this manuscript:

TMD	Temporomandibular disorder
PsA	Psoriatic arthritis
TMJ	Temporomandibular joint
CS	Central sensitization
DC/TMD	Diagnostic Criteria for Temporomandibular Disorders
csDMARD	Conventional synthetic disease-modifying antirheumatic drug
bDMARD	Biologic disease-modifying antirheumatic drug
tsDMARD	Targeted synthetic disease-modifying antirheumatic drug
mMASES	Modified Maastricht Ankylosing Spondylitis Enthesitis Scoring
PASI	Psoriasis Area and Severity Index
cDAPSA	Clinical Disease Activity Index for Psoriatic Arthritis
VAS	Visual analogue scale
CRP	C-reactive protein
TJC	Tender Joint Count
SJC	Swollen Joint Count
HAQ-DI	Health Assessment Questionnaire-Disability Index
PSQI	Pittsburgh Sleep Quality Index
HADS	Hospital Anxiety and Depression Scale
PsAID-12	Psoriatic Arthritis Impact of Disease
FACIT	Functional Assessment of Chronic Illness Therapy
BMI	Body mass index
CSI	Central Sensitization Inventory
PPT	Pressure pain threshold
SD	Standard deviation
IQR	Interquartile range
OR	Odds ratio
