# Supplementary material for: The Role of Central Sensitization and Emotional Comorbidities in Temporomandibular Involvement Among Patients with Psoriatic Arthritis
Source: Life (Basel). 2026 Apr 21;16(4):697. doi: 10.3390/life16040697 (PMC13118211; doi:10.3390/life16040697)
Supplement: Supplementary file 1 [file life-16-00697-s001.zip › life-4222290-supplementary.pdf]

Supplementary Material.

Table S1. Coefficients of logistic Model.

					95%CI	
**Variable**	$\hat{\beta}$	**EE**	**p**	**Odds-ratio**	**Lower**	**Upper**
Constant	-7.04	3.11	0.024	0.001	0.00	0.39
FACIT-F	0.02	0.05	0.685	1.02	0.93	1.12
Kinesiophobia	-0.03	0.05	0.545	0.97	0.89	1.06
CSI	0.11	0.03	<.001	1.11	1.05	1.18
Pain threshold	0.08	0.25	0.740	1.04	0.67	1.75
Pain_VAS	0.01	0.16	0.978	1.01	0.74	1.36
PSQI	-0.10	0.10	0.277	0.91	0.76	1.08
HADS_A	0.28	0.12	0.021	1.32	1.04	1.67
HADS_D	-0.18	0.13	0.166	0.84	0.65	1.09
enthesitis	0.06	0.12	0.602	1.06	0.85	1.33
TJC	0.13	0.14	0.342	1.14	0.87	1.48
SEX:(Male)	1.16	0.69	0.096	3.19	0.8	12.44
Biological Treatment (Yes)	-0.95	0.63	0.128	0.39	0.11	1.32

EE: Standard Error

Table S2.- Diagnosis of multicollinearity using the Variance Inflation Factor (VIF) for the model variables.

	**VIF**
FACIT-F	1.281
Kinesiophobia	2.943
CSI	1.630
Pain threshold	1.989
Pain_VAS	1.237
PSQI	1.718
HADS_A	2.134
HADS_D	2.506
enthesitis	2.819
TJC	1.484
SEX	1.445
Biological Treatment	1.185
